# Exercise as a Therapeutic Strategy to Improve Cerebrovascular Function and Cognition in Breast Cancer Survivors: A Scoping Review

**DOI:** 10.3390/jcm13247841

**Published:** 2024-12-22

**Authors:** Tahnee L. Downs, Eliza J. Whiteside, Joshua Denham, Dean E. Mills, Edward S. Bliss

**Affiliations:** 1 School of Health and Medical Sciences, University of Southern Queensland, Toowoomba, QLD 4350, Australia; 2 Respiratory and Exercise Physiology Research Group, University of Southern Queensland, Ipswich, QLD 4305, Australia; 3 Centre for Health Research, University of Southern Queensland, Ipswich, QLD 4305, Australia; 4 Centre for Future Materials, University of Southern Queensland, Toowoomba, QLD 4350, Australia

**Keywords:** cognition, cerebrovascular function, exercise, breast cancer, survivor

## Abstract

Breast cancer is the most diagnosed cancer globally. While the breast cancer prevalence continues to rise, so too do patient survival rates, thus resulting in a large survivor population. Up to 75% of this population report experiencing cancer-related cognitive impairment during their cancer journey, thus reducing their quality of survivorship. This review systematically evaluates the effect of physical activity and exercise training on cerebrovascular function and cognition in breast cancer survivors. Cross-sectional, intervention or observational studies that examined the effect of acute or chronic exercise training or physical activity levels on cerebrovascular function and cognition in female breast cancer survivors were searched for systematically. The 11 included studies were tabulated and described narratively. The included studies primarily focused on aerobic exercise training, while only four studies investigated the effect of resistance exercise training or concurrent training on cerebrovascular function and/or cognition in breast cancer survivors. Collectively, these studies provide preliminary evidence supporting the positive effect of exercise training on cerebrovascular function and cognition in breast cancer survivors, irrespective of their age, stage of breast cancer and treatment regimen. However, more research is required to comprehensively evaluate the effect of exercise training on cerebrovascular function and cognition in breast cancer survivors and the mechanisms leading to these potential improvements.

## 1. Introduction

In 2022, 2.3 million women were diagnosed with breast cancer [1,2]. Among women, breast cancer is the most commonly diagnosed cancer and the second leading cause of cancer death [3]. By the end of 2022, there were a total of 8.3 million women alive who were diagnosed within the past five years alone [2]. Although the breast cancer incidence continues to rise, so too does short-term and long-term survival. Globally, the average 5-year survival rate from breast cancer in high-income countries may be greater than 85% but can be lower than 40% in low–middle-income countries [4]. For example, in Australia, a high-income country, the current 5-year survival rate is 92% [5], while in Algeria, a lower–middle-income country, this drops to 38.8% [4]. The higher survival rates experienced in high-income countries are largely due to state-funded screening campaigns for early detection, combined with increasingly advanced breast cancer therapies, which have significantly reduced mortality and recurrence. Although many of these new therapies are effective in prolonging life, they can be associated with significant side effects and can have profound effects on the structure and function of the brain, which underlie cognitive dysfunction [6]. Indeed, 75% of breast cancer patients report cancer-related cognitive impairment (CRCI) during treatment and into survivorship [7]. This has created an unusually large population of survivors, with the majority suffering from CRCI and a reduced quality of life (QoL) [8]. Breast cancer survivors lose more disability-adjusted life years (the equivalent of one year of full health) than those of any other cancer, and cognitive impairment is a major contributing factor [9].

CRCI is poorly understood, as too are the mechanisms underlying the development of CRCI in breast cancer patients, despite several studies investigating the effect of breast cancer treatment regimens on cognition [10]. The potential mechanisms observed in animal models have been described previously [11]. These mechanisms include general oxidative damage resulting from increased oxidative stress both centrally and systemically; increased systemic inflammation and subsequent neuroinflammation; neurotoxicity, which is primarily focused on the effect chemotherapy has on reduced neurogenesis and brain volume; and an altered hormonal status, particularly reduced hypothalamic–pituitary–adrenal axis activity [11]. Interestingly, animal studies have also noted reductions in the cerebrovascular density and cerebral blood flow (CBF), with limited studies assessing this further in human cancer patients [11,12]. This is of interest as it has been established that cognitive decline is preceded by reduced cerebrovascular function, and both reduce concurrently throughout the ageing process [13,14,15]. A decline in cerebrovascular function and cognition has been observed in individuals without breast cancer, in particular those who have a chronic disease that is associated with reduced endothelial function, increased oxidative stress and chronic low-grade systemic inflammation [13,16,17]. These factors, when present in a chronic disease, further exacerbate the age-related decline in cerebrovascular function [13,16,17]. Essentially, a reduction in cerebrovascular function reduces the ability of the brain to acquire nutrient- and oxygen-rich blood to perform its functions, such as cognition, efficiently [13,16,17]. This reduced efficiency can be objectively measured through various techniques, including transcranial Doppler ultrasonography (TCD), magnetic resonance imaging (MRI), arterial spin labelling and positron emission tomography [18].

A recent study that investigated cerebrovascular function in breast cancer survivors reported that the CBF was lower compared to cancer-free, age-matched women (mean = 43 years) [19]. This confirmed the findings of two earlier studies that reported cerebrovascular dysfunction in breast cancer patients 1 month post-chemotherapy and another that reported a 9% higher prevalence of cerebrovascular microbleeds than in those without cancer 20 years post-diagnosis [20,21]. These findings are important because they suggest that the development of cognitive decline may be associated with diminished cerebrovascular function in women with breast cancer, which aligns with animal models.

Limited studies have assessed the connection between reduced cerebrovascular function and cognition, with only one study to date assessing both cerebrovascular function and cognition in breast cancer survivors [22]. The results of this study demonstrated that cerebrovascular function and cognition were reduced in breast cancer survivors (within five years of the end of their primary treatment; stages I–III) compared to cancer-free women matched by age (mean = 63 years) and body mass index (BMI; mean = 27.5 kg/m^2^). Specifically, breast cancer survivors had approximately 60% lower cerebrovascular responsiveness to physical stimuli and 37% lower cerebrovascular responsiveness to cognitive stimuli. In this study, cerebrovascular responsiveness was quantified using TCD by measuring the change in the mean blood flow velocity through the middle cerebral artery. Breast cancer survivors also had 13% lower total cognition than cancer-free women, as scored by the age-adjusted total compositive cognitive score of a validated, comprehensive cognitive battery. Hence, this study suggested that breast cancer, like other chronic diseases, may (a) further impair the perfusion of cerebral tissue in response to stimuli (i.e., vasodilator responsiveness) by impairing arteriolar endothelial function and (b) compromise the blood–brain barrier by impairing capillary endothelial function, thus contributing to cognitive impairment in breast cancer survivors compared to the general population [22]. Interestingly, the same study reported that higher exercise performance was positively associated with greater total cognition and cerebrovascular function. This has also been reported in individuals with metabolic syndrome [23], and exercise training has been demonstrated to improve cerebrovascular function and/or cognition in healthy older adults who are physically inactive [24,25] and in other chronic diseases, such as obesity [16], stroke [26], coronary artery disease [27] and Alzheimer’s disease [28]. These studies provide evidence that exercise training may either improve or maintain optimal cerebrovascular function, thus preventing or reducing the progression of cognitive impairment. Hence, physical activity and/or exercise training interventions may improve cerebrovascular function and cognition in breast cancer patients through a complex and multifaceted chain of events which reduce chronic inflammation and oxidative stress [13,22]. When these are reduced, endothelial function, arterial compliance and cardiovascular function may improve, which in turn promotes cerebrovascular function and the ability of the cerebrovasculature to provide increased CBF in response to increased neuronal metabolism, resulting in improved cognition [13,22].

Therefore, the purpose of this scoping review is to examine the effect of physical activity and exercise training on cerebrovascular function and cognition in female breast cancer survivors. Firstly, a review of CRCI and its aetiologies in breast cancer patients is provided. Secondly, a detailed discussion and critique of the evidence on the influence of exercise on cerebrovascular function and cognition in breast cancer survivors is provided. Finally, the potential mechanisms of the effect of exercise on cerebrovascular function and cognition in breast cancer survivors is discussed.

## 2. Materials and Methods

To identify existing studies specific to the topic being evaluated, PRISMA guidelines for scoping reviews were followed [29]. The literature was searched for systematically using PubMed (including MEDLINE), SCOPUS and EMBASE (OVID) up until 31 July 2023. For inclusion in this literature review, all articles must have been peer-reviewed, been written in English and included the use of exercise (aerobic, resistance/strength or multimodal) and must have utilised objective measures of cognition. Further, these studies must either have had a cross-sectional design or used physical activity or exercise training as an intervention. In this review, aerobic exercise training (AT) was defined as repetitive exercise training, which recruits large muscle groups and utilises energy produced via aerobic respiration, such as running, cycling, swimming and/or walking [30]. Resistance exercise training (RT) was defined as repeated bouts of muscle contraction against external resistance, with the expectation of increases in muscle strength, endurance and hypertrophy [31]. Concurrent exercise training (CT) was defined as the combination of at least two forms of exercise training, such as AT and RT [13]. The primary search terms used were ‘breast cancer and ‘exercise’ and any of the following additional terms: cerebral blood flow (CBF), cerebral blood velocity (CBV), cerebrovascular function, cerebral perfusion, cerebral volume, transcranial Doppler (TCD), magnetic resonance imaging (MRI), arterial spin labelling (ASL), near-infrared spectroscopy (NIRS) and cognition or cognitive function. All duplicate articles were removed. Articles were initially screened by TLD and ESB by the title, followed by the abstract and, if deemed suitable, were read in full and included below. Any conflicts regarding eligibility were resolved by a third reviewer (DEM). The reference lists of the suitable articles were also screened and included if deemed eligible. The selection and organisation of the included articles took place in a Microsoft Excel document file. The results of this search were tabulated and included the population (number of participants, age of participants, stages of cancer), intervention (physical activity and/or exercise training regimen describing the frequency, intensity, duration and level of supervision if provided), study design (analytical or observational) and outcomes (methods used to determine cognition and cerebrovascular function and the results obtained). The primary outcomes of these are discussed narratively.

## 3. Cognition and Cancer-Related Cognitive Impairment

Cognition is one of the most highly ordered and complex functions of the brain and reflects the ability of the brain to acquire, process, store and retrieve information in order to guide thoughts, actions and behaviours [32]. Therefore, intact cognition is fundamental in human living and sustaining a good QoL [33].

CRCI is characterised by a neuropsychological decline across various cognitive domains, including the processing speed, language, executive function and learning and memory (Table 1) [8]. CRCI may present as difficulties in concentration, thinking, multi-tasking, action planning, reasoning and problem solving and impaired perception and memory loss [34]. This decline can persist well into survivorship and is detrimental to a survivor’s QoL. However, not every cancer patient develops CRCI. Several factors are thought to increase the risk of CRCI development, including both non-modifiable and modifiable factors. Non-modifiable risk factors include age and a genetic predisposition [35]. Modifiable risk factors include the presence of co-morbidities, particularly cardiovascular disease and diabetes, as well as psychological (anxiety, depression and fatigue) and demographic and social factors (ethnicity, socioeconomic status and education) [11]. A recent scoping review reported cancer patients who belonged to racial and ethnic minority groups were at an increased risk of poorer cognitive outcomes and, therefore, CRCI [36]. However, the same study indicated that a lower education and socioeconomic status and sociocultural factors may influence the prevalence of CRCI. In addition, modifiable lifestyle factors, such as diet and physical inactivity, are risk factors for CRCI and, in developed, high-income countries such as Australia, physical inactivity is one of the most critical factors that promotes cognitive impairment [37]. This has also been reported by the World Health Organisation (WHO), who highlight that greater amounts of moderate to vigorous physical activity are associated with improvements in cognition, brain function and structure and a reduced risk of developing cognitive impairment [38].

Although the exact mechanisms by which CRCI occurs are poorly understood, several candidate mechanisms have been suggested. These are mostly based on findings in animal models. These include the inhibition of hippocampal neurogenesis, reduced white and grey matter, increased oxidative stress and chronic low-grade inflammation and decreased hypothalamic–pituitary–adrenal axis activity, brain vascularisation and cerebral perfusion [11]. Although the exact mechanisms underlying CRCI have not been elucidated, mounting evidence suggests a consistent association between cancer, cancer treatment and CRCI, and it is undoubtedly linked to changes in cerebrovascular function [8,56,57].

## 4. Cerebrovascular Function

Cerebrovascular function is the ability of the cerebrovasculature to perfuse the brain with blood and respond to increased metabolic demands and environmental changes, as well as ensure the CBF is constantly maintained [58]. The brain is one of the most metabolically active organs in the body, receiving 15–20% of the total cardiac output [59]. The brain does not store energy and, therefore, requires a constant controlled supply of nutrient- and oxygen-rich blood in order to perform its functions efficiently [17]. The supply of arterial blood to the brain via the cerebral circulation is referred to as the CBF, which is tightly regulated and increases concurrently with neuronal activity and metabolic demands [13]. The CBF can be determined through the blood viscosity, the extent of vasodilation and the cerebral perfusion pressure and is regulated by autoregulation (macrovascular function) or neurovascular coupling (microvascular function) [16]. Cerebrovascular autoregulation ensures the CBF is maintained during changes in the systemic blood pressure by modulating the vascular resistance applied to the macrovasculature [17]. Neurovascular coupling (NVC) is the complex interaction, or ‘coupling’, between neuronal activity and local haemodynamic changes, which ensures the metabolic demands of active neural tissues are met by the microvasculature [60].

The endothelium is a fundamental component in maintaining vascular homeostasis. It plays a key role in the regulation of vasomotor tone and, as a result, the systemic blood flow and blood pressure. In addition, the endothelium is a fundamental component in the regulation of long-term blood vessel haemostasis, inflammation, angiogenesis and numerous other functions that have been previously described [13]. These functions are largely governed by the release of the potent vasodilatory and vasoprotective hormone nitric oxide (NO). Endothelial nitric oxide synthase (eNOS) synthesises and releases NO onto juxtaposed smooth muscle, which relaxes, resulting in vasodilation and increased blood flow [61]. Thus, endothelial-derived NO is essential in maintaining cerebrovascular function and health. This assists in the acute regulation of vascular tone, and in doing so, the systemic blood flow and blood pressure. NO also supports the microvasculature to effectively respond to the brain’s metabolic demands [62], and it has been shown that endothelial-derived NO prevents reductions in the CBF, CBV and cerebral hypoperfusion [16]. The ability of the endothelium to synthesise and release NO decreases with age and conditions associated with chronic low-grade inflammation and oxidative stress, such as cancer [17,63]. These conditions exacerbate the development of endothelial dysfunction through the uncoupling of eNOS [62]. When eNOS is uncoupled, reactive oxygen species (ROS) are produced, instead of NO. ROS increase oxidative stress and further promote eNOS uncoupling and reductions in NO, which further induce endothelial dysfunction, resulting in impaired vascular function [16]. This reduces the ability of the cerebrovasculature to meet the metabolic demands of the brain, and it is unable to perform its functions efficiently, particularly cognition [17]. This inefficiency results in cognitive impairment, thereby indicating an important relationship between cerebrovascular function and cognition [13]. However, the mechanisms by which cognition and cerebrovascular function decline, particularly in breast cancer survivors, are poorly described.

## 5. Putative Effects of Cancer and Cancer Treatments on Cerebrovascular Function and Cognition

Derangements to the cerebrovascular mechanisms, as previously discussed, can lead to cerebral dysfunction, cerebral pathology and impaired cognition, and may promote the development of future neurodegenerative disease states [13]. Cerebrovascular function can be affected by various disease pathologies, including cancer [64]. The oxidative stress and chronic inflammatory state caused by cancer has significant repercussions on endothelial function, which is vital in maintaining the cerebrovascular structure and function [58]. The cancer disease state itself can impact brain health; however, cancer treatment can have profound effects on cerebrovascular function and is directly associated with impaired cognition [10].

### 5.1. Chemotherapy

Despite being extensively studied, the underlying mechanisms that drive chemotherapy-induced cognitive impairment are poorly described. Previous studies have demonstrated that chemotherapy induces vascular damage, including increased plaque formation, which promotes vascular stenosis. This, in turn, can reduce the cerebrovascular density, thus reducing the CBF [65]. This damage has been shown to persist for up to 20 years post-treatment, with breast cancer survivors treated with chemotherapy and radiotherapy possessing a lower total CBF and brain perfusion than cancer-free, age-matched women [19]. The decline in the total CBF and brain perfusion is likely attributable to a decline in hippocampal neurogenesis [19].

The endothelium is the first organ in contact with any intravenous chemotherapeutics and is damaged by this exposure, presenting as endothelial dysfunction [66]. Further, chemotherapeutics have been shown to reduce eNOS bioavailability and/or activation within the endothelium [63]. This loss can result in increased vascular inflammation and reduced cerebrovascular function and CBF, which ultimately results in impaired cognition [13]. The endothelium may never fully recover from the damage caused by chemotherapy, and this may explain the prolonged altered brain perfusion observed in survivors [19].

Chemotherapeutics are also heavily implicated in neuroinflammation, an inflammatory response within the brain due to the dysregulation of cytokines and chemokines [67]. Cytokines play an important role in cell signalling events which regulate immune and inflammatory responses, initiating or mediating several peripheral and neuronal immune cascades. They are also able to readily cross the blood–brain barrier (BBB). The BBB is composed of tightly joined endothelial cells which prevent toxins in the peripheral immune system from entering the brain. Most chemotherapeutics cannot cross the BBB. However, they induce increased levels of pro-inflammatory cytokines, which can cross the BBB. Several studies have demonstrated an increased concentration of cytokines following the administration of various chemotherapeutics [68,69,70]. This increase results in neuroinflammation and can change the structure and integrity of the BBB, which facilitates further cytokine crossing (deleterious to tight junctions) and can also permit the crossing of chemotherapy drugs themselves. Increased BBB permeability or ‘leakage’ has also been implicated in the pathogenesis of cognitive decline [71].

Chemotherapy can induce cognitive impairment. Several studies examining breast cancer populations have shown that chemotherapy negatively affects multiple cognitive domains including visual memory [46], verbal memory [40], attention [41], verbal recall [44], working memory and delayed recall [45], executive function [43] and the processing speed [39]. Indeed, a recent meta-analysis investigated the link between chemotherapy and cognitive decline in breast cancer survivors and found that chemotherapy significantly impaired the cognitive domains of memory and language, attention, executive function, the information processing speed and visual and verbal memory, compared to non-cancer controls [56]. A more recent systematic review of chemotherapy-induced cognitive impairment discussed the results of 30 studies (breast cancer accounted for 21 of the 30) [72]. The authors reported the cognitive domains most affected by chemotherapy were (in descending order of frequency) the processing speed, verbal learning and/or memory, attention, motor function and coordination, executive function, memory, the reaction speed, visual memory, the psychomotor speed, semantic fluidity, concentration, working memory and visuospatial abilities.

### 5.2. Radiotherapy

Similarly, radiotherapy can reduce vascular function by causing endothelial dysfunction. Depending on the dose and target organ, radiotherapy affects the endothelium in different ways and may have acute or chronic manifestations. Chronic radiotherapy effects are often observed in the cerebral circulation and can cause endothelial cell senescence. This is characterised by permanent cell cycle arrest and a pro-inflammatory secretory phenotype [73]. Senescence is a mechanism implicated in long-term vascular dysfunction [74]. In high doses, radiotherapy can deplete endothelial cell populations, thus promoting endothelial dysfunction leading to reduced vascular impairment [75]. Additionally, oxidative stress due to the decomposition of water molecules into ROS from ionising radiation promotes endothelial dysfunction and increases inflammation in the irradiated area [75].

Breast cancer survivors treated with radiotherapy exhibit decreased performance in verbal memory and memory recall testing compared to survivors not treated with radiotherapy [49]. Further, across multiple treatment modalities (chemotherapy, radiotherapy and chemotherapy and radiotherapy), breast cancer survivors demonstrate comparable cognitive impairment in the domains of learning and memory, attention, executive function, the information processing speed and visuospatial processing [76]. However, studies investigating radiotherapy-induced cognitive impairment are limited in that they are often confounded by the presence of other anti-cancer treatment modalities and rarely possess wholly chemo-naïve study populations.

### 5.3. Hormonal Therapy

Although significantly less studied than chemotherapy and radiotherapy, hormonal therapy has been shown to lead to organisational, activation, neurotropic and neuroprotective deficits [77]. Impaired cognition in the domains of speech, memory, vocabulary, and decision-making has been observed in breast cancer survivors post-endocrine therapy [52,54,78]. Additionally, hormonal therapy is posited to be a precursor to endothelial, and ultimately vascular, dysfunction, given the relationship between oestrogen and vasodilation [79]. Oestrogen is a vasodilator and hypotensive agent which promotes vasodilation by stimulating the release of endothelium-derived vasodilatory substances and decreasing the production of vasoconstrictive agents [80]. Similarly to radiotherapy, studies exploring the effect of hormonal therapy on cognition are confounded by prior or concurrent chemotherapy treatment, and as a result, study populations are modest.

### 5.4. Summary—Cancer Treatments and CRCI

Although typically associated with treatment, CRCI has also been observed in cancer patients prior to treatment commencement. These changes are summarised in Table 1. This suggests that the cancer disease state itself can also lead to cognitive impairment. The oxidative stress and chronic inflammatory state caused by cancer has significant repercussions for endothelial function, which is vital in maintaining the cerebrovascular structure and function [58]. Regardless, the mechanisms underpinning the effects of both cancer and anti-cancer treatments in the development of CRCI have been poorly described, as longitudinal studies are lacking. Given that the population of breast cancer survivors is rapidly increasing, this question is becoming increasingly pertinent and could prove invaluable in informing future studies seeking to attenuate CRCI. Regardless of the exact cause of CRCI, it is well established that cognitive decline is preceded by a decline in cerebrovascular function [13].

## 6. Results

**Figure 1:**
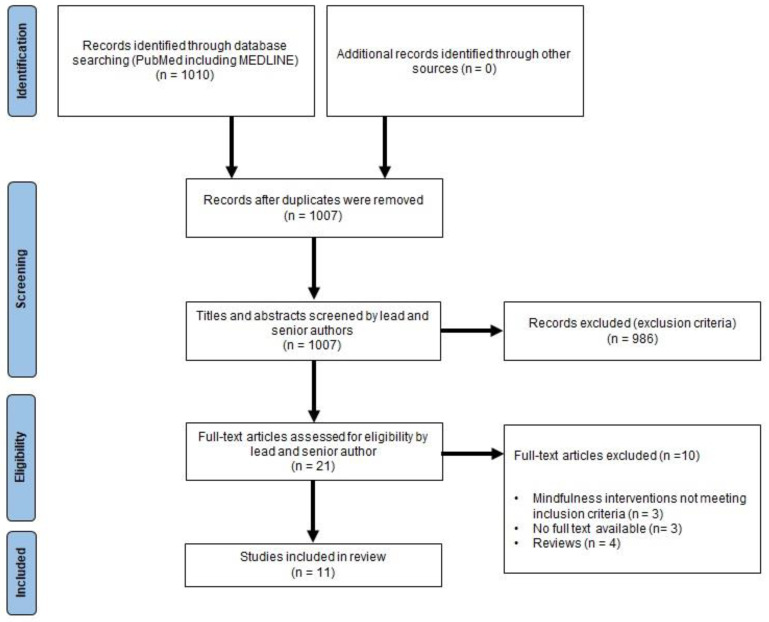
PRISMA flow diagram.

There were 1010 studies identified from the initial search using the PubMed and Medline databases. No other records were identified through other sources, and only three duplicate articles were removed by the lead author (TLD). Both the lead and senior author (ESB) screened the remaining 1007 titles and abstracts and excluded all articles that were not relevant to the topic and research question. Only 21 articles remained, and both the lead (TLD) and senior (ESB) authors reviewed the full-text articles for inclusion. Following this, 11 articles remained and were subsequently included in this study. The results of this process can be seen in Figure 1. The results of all the studies included are tabulated in Table 2 and described narratively below. The results described in Table 2 were reported to be statistically significant (*p* < 0.05) unless otherwise indicated (i.e., no change reported).

Only eight of the included studies conducted chronic exercise training, assessing its effect on cognition and/or cerebrovascular function in breast cancer survivors. These studies also assessed cognition and/or cerebrovascular function both at baseline and upon the completion of the exercise training intervention. Of these studies, only five reported studies reported exercise adherence. This is described in Table 2.

## 7. Exercise for Cancer Patients

The benefits of exercise for overall health and wellbeing in cancer patients are well described [81]. Specifically, exercise training attenuates the severity of cancer treatment-related side effects such as fatigue, nausea and a loss of lean mass and bone density, as well as impacts on physical and psychological function [82]. Epidemiological studies have also highlighted that exercise has a protective role in improving breast cancer patient outcomes by reducing recurrence and mortality [83]. Further, AT, RT and CT have been shown to improve symptoms related to anxiety and depression in breast cancer patients [84,85,86]. This further adds evidence to why exercise training should be considered as an intervention to improve cognition and cerebrovascular function. Exercise training may also improve impaired cerebrovascular function and prevent or slow the progression of cognitive decline in those who have survived cancer. This may be achieved by increasing and maintaining cerebral perfusion and the cognitive capacity in at-risk populations, such as those suffering from breast cancer [16]. Exercise training has been shown to be an effective treatment for age-related cerebrovascular and cognitive decline. However, studies which comprehensively investigate this effect in breast cancer survivors are limited (see Table 2).

**Table 2:** Summary of studies that have measured the effect of exercise on cognition and cerebrovascular function.

Reference	Study Design	Participant Description	Primary Outcome	Method Used to Measure Primary Outcome	Results of Primary Outcome	Other Significant Results and Reported Exercise Adherence
**Effect of physical activity on cognition**						
**Marinac et al. (2015) [87]**	Randomised control trial Acute unsupervised physical activity at defined intensities (sedentary, LIPA and MVPA) 7-day intervention	Female breast cancer survivors (*n* = 136) All ages (mean of 62.6 years) Stages I–III <5 years post-diagnosis	Cognition	NeuroTrax comprehensive testing suite	Time spent doing MVPA was associated with ↑ information processing speed	None reported
**Ehlers et al. (2017) [88]**	Cross-sectional acute unsupervised AT 7 days of MVPA	Female breast cancer survivors (*n* = 299) >21 years old (mean of 57.5 years) All stages of breast cancer	Cognition	Cognitive battery (Flanker, mazes, task switch, TMT-B, SSP, SWM, swap test)	Time spent doing MVPA was associated with ↑ executive function ↑ working memory	↓ fatigue
**Effect of a single bout of exercise on cognition**						
**Salerno et al. (2019) [89]**	Randomised crossover trial Acute supervised AT 2 × 30 min sessions in total Session 1: moderate intensity AT Session 2: seated rest	Female breast cancer survivors (*n* = 27) 30–60 years old (mean of 49.1 years) Stages DCIS-IIIB Completed primary treatment	Cognition	Cognitive battery (LCT, SWM)	↑ processing speed ↑ spatial working memory	None reported
**Effect of chronic exercise on cognition**						
**Miki et al. (2014) [90]**	Randomised control trial Supervised speed feedback therapy with a cycle ergometer 4 × 5 min sessions One session per week for 4 weeks	Female breast cancer survivors (*n* = 21) >65 years old (mean of 74.2 years)	Cognition	FAB	↑ frontal cognitive function	None reported
**Galiano-Castillo et al. (2017) [91]**	Randomised controlled trial 3 × 90 min sessions of CT per week for 8 weeks	Female breast cancer survivors (*n* = 81) All ages (mean of 48.3 years) Stages I–IIIA Completed primary treatment	Cognition Exercise capacity	ACTs TMT-A and TMT-B	↑ short-term memory ↑ attention span ↑ information processing speed	↑ distance in 6MWT 94% exercise adherence
**Campbell et al. (2018) [92]**	Randomised control trial 24-week moderate intensity AT 4 sessions per week, totalling 150 min/week Session 1 and 2: supervised, 45 min Sessions 3 and 4: unsupervised in home, 30 min	Female breast cancer survivors (*n* = 19) 40–65 years old (mean of 52.4 years) Stages I–IIIA Self-reported CRCI >3–36 months post-diagnosis	Self-reported cognition	Self-reported cognitive function (FACT-Cog) Cognitive battery (HVLT-R, COWAT, Stroop test) MRI	No difference after AT	↓ brain activation in cingulate cortex and superior frontal gyrus 88% exercise adherence (supervised sessions) 87% exercise adherence (unsupervised sessions)
**Hartman et al. (2018) [93]**	Randomised control trial 12-week unsupervised, moderate intensity AT 150 min/week of walking	Female breast cancer survivors (*n* = 87) 21–85 years old (mean of 57.2 years) >5 years post-diagnosis	Cognition	NIH Toolbox PROMIS	↑ processing speed	None reported
**Mijwel et al. (2018) [6]**	Randomised control trial 16-week supervised intervention 2 × 60 min sessions per week (1) AT-HIIT OR (2) RT-HIIT	Female breast cancer survivors (*n* = 240) 18–70 years old (mean of 53.2 years) Stages I–IIIA	CRF	PFS	RT-HIIT: cognitive function unchanged Control: ↓ cognitive function	RT-HIIT: ↓ CRF (total CRF, behaviour and sensory) ↓ symptom burden 83% exercise adherence AT-HIIT: ↑ emotional functioning Symptom burden remained stable 75% exercise adherence
**Peterson et al. (2018) [94]**	Randomised control trial 12-week supervised intervention (1) Computer-based cognitive exercise OR (2) 3 × 30 min/week AT cycle ergometry OR (3) Combination of (1) and (2)	Male and female cancer survivors (*n* = 28) <69 years old (mean of 57.9 years) All stages Breast cancer (*n* = 14)	Cognition	Cognitive battery (WMS-IV; BCOG; TMT-A; WAIS-IV; LNS; CD; TMT-B; LMI 1; LMI 11; COWAT; BD) NeuroActive ^®^ cognition training software	AT: ↑ logical memory, delayed recall, block design scores and letter–number sequencing scores	None reported
**Koevoets et al. (2022) [95]**	Randomised control trial 24-week CT intervention (1) Supervised session: 120 min RT+AT, plus (2) Unsupervised session: 120 min AT	Female breast cancer survivors (*n* = 181) 30–75 years old (mean of 52.1 years) >2–4 years post-diagnosis Stages I–III	Memory	HVLT-R, ACS	No difference after CT	↓ fatigue ↓ depression severity ↑ QoL ↑$\dot{V}$O_2_peak ≥80% exercise adherence
**Effect of exercise on cerebrovascular function**						
**Northey et al. (2019) [7]**	Randomised control trial 12-week HIIT or moderate intensity AT HIIT: supervised, 3 × 30 min/week, moderate intensity AT: unsupervised, 3 × 30 min/week	Female breast cancer survivors (*n* = 17) 50–75 years old (mean of 63.2 years) ≤24 months post-diagnosis	Cognition	TCD CogState battery	HIIT: ↑ episodic memory, executive function, working memory ↑ MCA_V_ and CVR to hypercapnia MOD: ↑ episodic memory ↑ MCA_V_ and CVR to hypercapnia	↑$\dot{V}$O_2_peak 79% exercise adherence

Abbreviations: LIPA, light-intensity physical activity; MVPA, moderate–vigorous physical activity; AT, aerobic exercise training; TMT-A, Trail-Making Task A; TMT-B, Trail-Making Task B; SSP, Spatial Span Task; SWM, Spatial Working Memory Task; DCIS, ductal in situ carcinoma; LCT, Letter Comparison Task; FAB, Frontal Assessment Battery; CT, concurrent exercise training; ACTs, Auditory Consonant Trigrams; 6MWT, six-minute walk test; FACT-Cog, Functional Assessment of Cancer Therapy; HVLT-R, Hopkins Verbal Learning Task—Revised; COWAT, Controlled Oral Word Association Test; MRI, magnetic resonance imaging; PROMIS, Patient-Reported Outcomes Measurement Information System; CRF, cancer-related fatigue; PFS, Piper Fatigue Scale; RT, resistance exercise training; HIIT, high-intensity interval training; WMS-IV, Wechsler Memory Scale, Fourth Edition; BCOG, Brief Cognitive Scale; WAIS-IV, Wechsler Adult Intelligence Scale, Fourth Edition; LNS, Letter and Number Sequencing Test; CD, Coding Test; LMI I, Logical Memory Test 1; LMI II, Logical Memory Test 2; BD, block design test; ACS, Amsterdam Cognition Scan; $\dot{V}$O_2_peak, volume of oxygen uptake during peak exercise; TCD, transcranial Doppler ultrasonography; MCA_V_, middle cerebral artery blood velocity; CVR, cerebrovascular responsiveness.

### 7.1. Effect of Physical Activity on Cognition in Breast Cancer Survivors

Two cross-sectional studies have examined the association between cognition and physical activity in breast cancer survivors. Marinac et al. (2015) [87] examined the association between physical activity levels and cognitive function. Participants wore an accelerometer for seven consecutive days in order to measure the daily time spent on activities of defined intensities (sedentary, light-intensity physical activity and moderate–vigorous physical activity). Participants completed the NeuroTrax comprehensive testing suite, a 45 min computerised cognitive battery designed to sample a range of cognitive domains to assess overall cognitive functioning. There was a positive association between the time spent on moderate–vigorous physical activity and the information processing speed. Specifically, 10 min per day of moderate–vigorous physical activity was associated with a 1.35-point higher score for the information processing speed. However, there were no statistically significant associations between light-intensity physical activity and cognition. The authors suggested that physical activity interventions which specifically target moderate–vigorous physical activity may be able to enhance aspects of cognitive function among breast cancer survivors.

Ehlers et al. (2017) [88] performed a similar cross-sectional study, using an American sample of female breast cancer survivors to examine the association between physical activity levels and cognitive function. Participants wore an accelerometer for seven consecutive days in order to measure their average daily minutes of moderate-to-vigorous activity and completed a cognitive battery to assess executive function and working memory. Daily participation in moderate-intensity physical activity was positively associated with increased executive function and working memory and decreased fatigue. A limitation of this study exists in its study population, which was generally well-educated, affluent and white and may not be reflective of other disadvantaged populations (e.g., other races, a low socioeconomic background, regional/rural, etc.). Inclusion criteria included the ownership/possession of an iPad (in order to complete the cognitive battery).

Despite the limited number of cross-sectional studies, the findings suggest that moderate- to vigorous-intensity exercise may be necessary to promote cognitive functioning in breast cancer survivors, whereas a light intensity could be insufficient.

### 7.2. Effect of a Single Bout of Exercise on Cognition in Breast Cancer Survivors

Consistent with findings in the general population, a single bout of aerobic training seems to improve cognitive function in breast cancer survivors. Salerno et al. (2019) [89] investigated the effects of a single bout of moderate-intensity AT on cognitive performance in female breast cancer survivors with stages ranging from a ductal carcinoma in situ, the earliest form of breast cancer, to IIIB cancer who had completed their primary treatment course. Using a repeated measure, crossover design, participants completed two AT sessions on two separate days in a counter-balanced order. These sessions comprised 30 min of moderate-intensity treadmill walking and 30 min of seated rest and the completion of a cognitive battery before and after each session. The study aimed to observe the cognitive performance of participants before, and immediately after, exercise. There were improvements in the processing speed and spatial working memory immediately post-exercise but no changes from pre- to post-seated rest. The authors suggested that the findings of these acute interventions demonstrated a positive association between AT and improved cognition in a breast cancer population.

Thus, the only trial to examine the effect of a single bout of aerobic training on cognition in breast cancer survivors indicated that at least moderate-intensity walking leads to short-term improvements in cognitive performance, which seems to be consistent with cross-sectional findings.

### 7.3. Effect of Chronic Exercise on Cognition in Breast Cancer Survivors

While short-term cognitive improvements are elicited by a single bout of moderate-intensity AT, regular long-term training is required for sustained health benefits. Miki et al. (2015) [90] conducted a four-week randomised controlled trial to compare the effects of supervised speed feedback therapy and cycle ergometer training undertaken in 4 × 5 min sessions once a week for 4 weeks (intervention group) compared to routine life activities without any rehabilitation intervention (control group) on cognitive function in elderly breast cancer patients. Cognition was assessed using the Frontal Assessment Battery (FAB), which was developed as a short bedside cognitive and behavioural battery to assess frontal lobe function across six domains. Speed feedback therapy was safe and well tolerated by participants, but the level of intensity and exercise prescription in this study was not made evident. The mean FAB score for the intervention group was higher than that of the control group at week four; however, this change was small. Despite this, the authors suggested that a small increase in FAB scores can lead to an improvement in the instrumental activities of daily living in elderly breast cancer survivors in a relatively short timeframe.

Galiano-Castillo et al. (2017) [91] investigated the effect of an eight-week at-home, internet-based, tailored exercise programme compared to usual care (control group) to improve the functional capacity and cognition among breast cancer survivors. The intensity of exercise in this study was not clearly defined. Participants completed three 90 min CT sessions per week for 8 weeks. Cognition was assessed using Auditory Consonant Trigrams (ACTs), which assess short-term memory, the attention span and the information processing capacity, as well as the Trail-Making Test (TMT), parts A and B, immediately following study completion. Relative to controls, the CT group displayed higher total ACT scores. However, no between-group difference was displayed in the TMT. Thus, the authors posit that a broad-reaching, internet-based exercise programme may achieve improvements in some cognitive domains; however, it may also have a maintenance effect on these improvements in breast cancer survivors.

Campbell et al. (2018) [92] investigated the effects of a 24-week AT intervention on cognition in female breast cancer survivors with stage I–IIIA cancers who were three months to three years post-diagnosis, compared to a control group. Participants completed 150 min per week of moderate-to-vigorous AT, consisting of two 45 min supervised sessions in a research gym and two additional 30 min unsupervised sessions. Cognition was assessed through self-reported measures, an objective cognitive battery and functional MRI (fMRI) both at baseline and upon study completion. No statistically significant improvements were reported in either self-reported cognitive dysfunction or performance in the neuropsychological test battery between the exercise cohort and control group. Similarly, the fMRI results did not reach significance between groups. However, the exercise cohort showed less activation in the cingulate cortex (limbic lobe) and superior frontal gyrus (frontal lobe). The cingulate cortex is implicated in emotion processing, reward-based decision-making, the interpretation of motor commands, visuospatial processing, imagination and the formation and consolidation of episodic memory [96], whereas the dominant (left) superior frontal gyrus is a key component in the neural network of working memory and spatial processing and the non-dominant (right) superior frontal gyrus is involved in impulse control [97]. Hence, this may suggest that less effort was required in order to facilitate the same level of cognitive performance as that observed prior to commencing the exercise intervention, as well as improved activation efficiency in these areas. The authors posited that this finding may indicate that exercise affects greater cognitive efficiency, which may produce a compensatory effect towards underlying cognitive deficits.

Hartman et al. (2018) [93] investigated the effects of a 12-week moderate intensity AT programme on self-reported and objective cognitive measures in female breast cancer survivors more than five years post-diagnosis compared to a waitlist control group. Participants were given a goal of achieving 150 min of moderate-intensity AT per week for 12 weeks and at baseline were supervised on a ten-minute walk at 65–75% of their maximum heart rate in order to demonstrate the level of exertion and pace-per-mile required to reach a moderate intensity. The National Institute of Health (NIH) Toolbox was used to objectively measure cognition, and the Patient-Reported Outcomes Measurement Information System (PROMIS) was used to assess self-reported cognition following the completion of the intervention. There was no change in all the tests comprising the NIH Toolbox following AT, except for the Oral Symbol Digit Score, which is responsible for measuring the processing speed. There were also no changes in self-reported cognition following AT. Cognition may not have improved in this study as the majority of the exercise intervention was largely unsupervised, with limited recording of how much and what type of exercise the participants performed over the 12-week period.

Mijwel et al. (2018) [6] conducted a 16-week exercise intervention with breast cancer survivors with stage I to IIIA cancers, examining the effect of adding high-intensity interval training (HIIT) to either AT or RT. The primary objective was to reduce cancer-related fatigue (CRF), and cognitive function was not assessed using a standard or validated measure. However, CRF and CRCI are closely intertwined. CRF is defined as a distressing, persistent, subjective sense of physical, emotional and/or cognitive tiredness or exhaustion. The self-administered 22-item Piper Fatigue Scale (PFS) was used to assess CRF, which covers four dimensions of fatigue, including 6 items which directly assess cognitive CRF. The addition of HIIT to RT improved self-reported cognitive problems. Cognitive function, which was self-assessed using the PFS, was unchanged for the RT–HIIT group from the baseline to study completion, compared to declines shown in the control group.

Peterson et al. (2018) [94] conducted a 12-week intervention investigating the effects of supervised AT compared to, and in combination with, computer-based cognitive training (COG) on cognition in older male and female cancer survivors. Female breast cancer survivors represented 50% of the study population, but no sub-analysis was performed on this group. Participants in the AT group completed three 60 min sessions per week, comprising 30 min of cycling and 30 min of flexibility training, while the COG group completed cognitive training via NeuroActive ^®^ cognition training software. An additional group completed both interventions (i.e., AT and COG). Participants who undertook AT showed increased performance in verbal learning and memory (logical memory, delayed recall), perceptual reasoning (block design test) and working memory, executive function and attention (letter and number sequencing). However, cognition was not improved in the participants who undertook COG and combined AT and COG. The group who completed AT and COG received both interventions simultaneously, where participants cycled at a target heart rate (between 55 and 65% of their heart rate reserve) while completing cognitive exercises. The authors highlighted that participants reported that receiving both physical and psychological stimuli simultaneously was overwhelming. This may indicate that the intervention was perceived as a burden by participants and may explain the poor results obtained for the group receiving both AT and COG.

Finally, Koevoets et al. (2022) [95] investigated the effects of 24-weeks of CT on cognition in a female breast cancer population with stage I–III cancer who were two to four years post-diagnosis, compared to a control group. Inclusion criteria included treatment with (neo)adjuvant chemotherapy and self-reported cognitive decline. The authors specifically focused on memory performance, which is a common complaint in chemotherapy-treated survivors, and this was assessed using the Revised Hopkins Verbal Learning Test. Secondary outcomes included objectively measured cognition assessed using an online test battery and self-reported cognition using a validated symptom assessment inventory (the MD Anderson Symptom Inventory for Multiple Myeloma). The exercise intervention consisted of 120 min of CT per week, supervised by a physiotherapist. Each session consisted of 20–25 min of AT and 20–25 min of RT. In addition, participants completed 120 min per week of AT (Nordic walking or powerwalking) at 55–65% of their heart rate reserve. There were no improvements in memory performance or objective cognitive measures following CT, but there were improvements in self-reported cognition, fatigue, depression severity and QoL, which were assessed following the intervention.

### 7.4. Effect of Exercise on Cerebrovascular Function and Cognition in Breast Cancer Survivors

Currently, only one study has investigated the effect of exercise on both cognition and cerebrovascular function in breast cancer survivors. This is important as both functions contribute to overall brain health and should be measured together in order to holistically determine the effects of exercise on overall brain health [13]. Northey et al. (2019) [7] investigated the effects of 12 weeks of either HIIT or moderate-intensity AT in female breast cancer survivors two years post-diagnosis. Participants completed three 30 min sessions per week of either HIIT or moderate-intensity AT in order to assess the effects on cognition, using the CogState cognitive battery, and cerebrovascular function, by assessing the resting middle cerebral artery (MCA) blood velocity and cerebrovascular reactivity to hypercapnia. HIIT elicited improvements in episodic memory, executive function and working memory, whereas moderate-intensity AT improved performance in episodic memory only. Further, the MCA blood velocity and cerebrovascular reactivity to hypercapnia (7% carbon dioxide, CO_2_) were higher in both exercise interventions than the control, with a larger size effect observed in the HIIT group. Although both exercise interventions elicited improvements compared to the control group, the authors posited that HIIT is a superior exercise intervention to moderate AT in improving cognitive outcomes in a breast cancer population. They also suggested that the improvement in cognition and cerebrovascular responsiveness to hypercapnia were probably due to improvements in endothelial function but indicated that further research is required to test this. However, the conclusions of the authors are limited due to the small participant number in each group.

Collectively, these studies provide preliminary evidence supporting the positive effect of exercise on cerebrovascular function and cognition in breast cancer survivors. However, two studies did not observe improved cognition. These results may be explained by potentially inappropriate research methodologies, including unsupervised exercise and self-reported cognition. The majority of studies observed improvements in cognition, while only a single study measured and observed improvements in cerebrovascular function following exercise. Few, if any, of these studies also indicate the potential mechanism/s by which exercise may improve cognition in women with breast cancer, thus highlighting another research gap.

### 7.5. Potential Mechanisms of the Effect of Exercise on Cerebrovascular Function and Cognition

The mechanisms by which exercise improves cerebrovascular function and cognition are poorly understood, particularly in breast cancer. The results of several clinical trials in ageing, sedentary adults suggest that higher amounts of physical activity are associated with improved cognition and a decreased risk of cognitive decline [98]. In contrast, physical inactivity is associated with decreased hippocampal neurogenesis and a loss of hippocampus volume, as well as decreased neurogenesis in the cerebellum and a loss of grey matter volume and cortical thickness [99]. Additionally, poor vascular health is positively associated with cognitive decline [13].

As previously discussed, cerebrovascular function facilitates brain perfusion and describes the ability of the cerebrovasculature to respond to increased neuronal metabolic demands and environmental changes (physical and chemical) and ensures the CBF is constantly maintained for the brain to perform its functions efficiently. Optimal cerebrovascular function requires optimal vascular health. Thus, a potential mechanism responsible for the effect of exercise on cerebrovascular function and cognition could be the beneficial effect it has on vascular function and, more specifically, endothelial function. The ability of the endothelium to synthesise and release NO is of paramount importance in CBF regulation to ensure the brain is adequately perfused to perform cognitive functions efficiently in response to either physical or psychological stimuli. Endothelial-derived NO reduces inflammation and oxidative stress, improves patency and vasomotor tone and maintains the capillary density [13]. Hence, further studies with an enhanced research methodology and increased participant numbers are required in order to ascertain a dedicated mechanism of action. Regardless of knowing the exact mechanisms involved, both cerebrovascular and cognitive function can be effectively measured and assessed in populations at risk of developing cognitive decline, including in breast cancer survivors.

## 8. Conclusions

Breast cancer is the most diagnosed cancer globally and its prevalence continues to rise. As a result, breast cancer research has been studied extensively. This has led to the development of highly advanced anti-cancer therapies which are able to increasingly prolong life. However, these therapies can be toxic and can cause significant off-target effects on the structure and function of the brain, which underlie the development of CRCI. This has resulted in an unusually large population of survivors, the majority of which are suffering from CRCI. This unique situation has prompted researchers to shift their focus from solely developing new anti-cancer therapies towards developing ways to improve the QoL. Simply put, researchers are not just focused on how long survivors live, but rather how well they live.

While the exact mechanisms underlying CRCI are not well understood, this impairment can persist into survivorship and may significantly impact the QoL in this population. Breast cancer and its treatments have significant repercussions on endothelial function, which results in a decline in cerebrovascular function, as the metabolic demands of the brain are not being met. Therefore, the brain is unable to perform its functions efficiently, which leads to cognitive impairment and highlights the interrelatedness of both functions in overall brain health.

Results from several clinical trials investigating exercise as a potential intervention to improve impaired cerebrovascular function and prevent, or slow, cognitive decline, are encouraging. These studies provide preliminary evidence supporting the positive effect of exercise on cerebrovascular function and cognition in breast cancer survivors; however, they possess limitations. Firstly, most of these studies have primarily focused on investigating the effect of AT interventions and have rarely incorporated any RT or conducted CT. Hence, future studies should aim to incorporate CT in order to exploit the combined individual benefits of both AT and RT for cerebrovascular function and cognition that have been described in other at-risk populations [13,100]. Secondly, most existing studies failed to measure both cerebrovascular function and cognition in unison, which is important given the interrelatedness of these functions. Therefore, future studies should measure both cerebrovascular function and cognition in unison, as both contribute to overall brain health. Thirdly, the one study which did measure cerebrovascular function did not measure cerebrovascular responsiveness to psychological stimuli (NVC). NVC is a key regulator of the CBF and reflects the complex interaction between neuronal activity and local haemodynamic changes. Hence, cerebrovascular responsiveness to both physical (physiological and/or chemical) and psychological stimuli should be assessed in order to better reflect the interrelatedness of cerebrovascular function and cognition and provide a holistic measurement of overall brain health. Fourthly, multiple studies incorporated self-reported cognition as either a primary or secondary study outcome. Self-reported cognitive tests do not display discriminant validity and are susceptible to the influence of numerous confounding variables, such as personality traits and self-presentational biases [101]. Research investigating the validity of self-reported cognition suggests individuals have only limited insight as the correlations between self-reported measures and objective cognitive performance measures are rarely significant [102]. Finally, a limitation of current studies exists due to inconsistent methodology. Study populations are small, non-diverse and represent varying cancer stages with significant differences in age groups. There is a lack of standardisation in the exercise protocol design and duration, varying definitions of exercise intensity and the inconsistent reporting of exercise adherence, if any. There are also unclear definitions of how randomisation and control selection occurred and the long-term follow-up of the efficacy of physical activity and exercise training interventions on cognition and cerebrovascular function is lacking. This can make comparisons between studies and the efficacy of physical activity and exercise interventions on cognition and cerebrovascular function challenging in some cases. Therefore, future research which addresses these limitations may provide important insight into how exercise training and physical activity may reduce the CRCI prevalence and improve the quality of survivorship in breast cancer patients.

## Figures and Tables

**Table 1:** Summary of the cognitive domain changes during breast cancer treatment.

Cancer Treatment	Effects of Treatment on Cognition	References
**Chemotherapy**	↓ verbal recall ↓ verbal memory ↓ verbal fluency ↓ working memory ↓ delayed recall ↓ attention and inhibitory control ↓ processing speed ↓ executive function ↓ visual memory	[39,40,41,42,43,44,45,46]
**Radiotherapy**	↓ verbal memory and delayed recall ↓ processing speed ↓ executive function ↓ working memory	[43,47,48,49]
**Hormonal therapy**	↓ learning ↓ verbal fluency ↓ language ↓ processing speed ↓ executive function ↓ verbal memory	[50,51,52,53,54,55]

## Data Availability

Data sharing is not applicable to this article as no datasets were generated or analysed during the current study.
